# The Posthumous Works of Sir James Y. Simpson, Bart

**Published:** 1871-04

**Authors:** 


### The Posthumous Works of Sir James Y. Simpson, Bart.

By a special arrangement with the Messrs. Black, of Edinburgh, and with the consent of the Executors of the late Sir James Y. Simpson, his posthumous works will be published in this country by Messrs. D. Appleton & Co., of New York. These works embrace in three volumes:

*Select Obstetric and Gynæcological Works.*—Edited by J. Watt Black, M. D., Physician Accoucheur and Lecturer on Midwifery and Diseases of Women and Children to Charing Cross Hospital, London.

*Anæsthesia, Hospitalism, etc., etc*—Edited by Sir Walter Simpson, Bart.

*The Diseases of Women.*—Edited by Alexander Simpson, M. D., Professor of Midwifery in the University of Edinburgh.

